# Psychosocial Risks in Spanish Nursing: Relationship With Stability and Working Conditions

**DOI:** 10.3389/ijph.2025.1608330

**Published:** 2025-05-20

**Authors:** Ángela Narbona-Gálvez, Juan Gómez-Salgado, Carlos Ruiz-Frutos, Diego Ayuso-Murillo, Guadalupe Fontán-Vinagre, Javier Fagundo-Rivera, Rocío Palomo-Gómez, Azahara Ruger-Navarrete, Regina Allande-Cussó

**Affiliations:** ^1^ School of Doctorate, University of Huelva, Huelva, Spain; ^2^ Department of Sociology, Social Work and Public Health, Faculty of Labour Sciences, University of Huelva, Huelva, Spain; ^3^ Escuela de Posgrado, Universidad de Especialidades Espíritu Santo, Espíritu Santo, Guayaquil, Guayas, Ecuador; ^4^ General Nursing Council of Spain, Madrid, Spain; ^5^ Spanish Institute for Nursing Research, Madrid, Spain; ^6^ Centro Universitario de Enfermería Cruz Roja, University of Seville, Seville, Spain; ^7^ Department of Obstetrics, Hospital La Línea de la Concepción, Andusian Health Service, Cádiz, Spain; ^8^ Department of Nursing, Faculty of Health Sciences of Ceuta, University of Granada, Granada, Spain; ^9^ Department of Nursing, Faculty of Nursing, Physiotherapy and Podiatry, University of Seville, Seville, Spain

**Keywords:** psychosocial risk factors, nurses, occupational stress, occupational health, working conditions

## Abstract

**Objectives:**

Psychosocial risks significantly affect the physical and mental health of workers, especially in the healthcare sector. This study assesses psychosocial risks in a sample of Spanish nurses by applying the ISTAS_ENFERMERÍA questionnaire.

**Methods:**

A descriptive cross-sectional study was conducted with 2,765 nurses working in Spain. Sociodemographic, occupational, and psychosocial variables were assessed using the ISTAS_ENFERMERÍA questionnaire. Data were collected through a digital form distributed through social networks and professional channels.

**Results:**

Significant differences were found in ISTAS_ENFERMERÍA scores according to socio-demographic and employment variables. Younger age and job instability were associated with higher levels of stress, while stable contracts were correlated with lower perceived risks.

**Conclusion:**

The study highlights the relationship between psychosocial risks and socio-demographic and occupational factors. Youth and job instability are linked to higher levels of stress, while job stability reduces these risks. Interventions to improve working conditions and support younger professionals are recommended.

## Introduction

Occupational safety and health (OSH) is a core domain in occupational risk management, which focuses on the prevention, identification, assessment, and control of risks that can arise both inside and outside the workplace [1]. It has become increasingly important due to the worrying rise in the number of occupational accidents and diseases, with 2.9 million deaths reported in 2019, of which nearly an 11% were caused by accidents and the rest by work-related diseases [2].

At the global level, occupational risks pose a major health challenge, with some studies estimating that between 5% and 7% of deaths worldwide are linked to risks in the work environment [3]. In example, occupational diseases such as silicosis and asbestosis remain common in developing countries, which emphasises the urgent need to improve regulation and labour practices [4].

In this context, psychosocial risks have gained importance and are described as those circumstances in the work environment that can negatively impact the physical and mental health or general wellbeing of workers. These risks include factors such as stress, the type of activities performed, the dynamics between colleagues and superiors, and the organisational environment in general, all of which can affect the ability of employees to adequately cope with the demands of the job [5].

In the field of health professionals, workers face numerous occupational risks that affect their physical and mental wellbeing, such as musculoskeletal injuries, exposure to hazardous substances, and violence at work, which are in turn compounded by stress and burnout [6]. The World Health Organization (WHO) estimates that globally, around 59 million health professionals suffer from work-related accidents and diseases, accounting for approximately 4% of the global Gross Domestic Product (GDP) [7].

In nursing, occupational risks are diverse and impact both the physical and mental health of professionals, ultimately compromising the quality of patient care [8]. Among these risks, psychosocial factors stand out, including high work demands, an intense work pace, and the overlap of professional and family responsibilities—a situation known as double presence. Additionally, the emotional demands of constant interaction with patients in critical conditions further contribute to these risks [9].

These factors intensify psychological exhaustion, increasing the likelihood of stress and burnout. As a result, both the wellbeing of healthcare staff and the standard of care they provide are negatively affected [10]. Recent studies highlight the prevalence and impact of psychosocial risks among nurses, particularly during health emergencies like the COVID-19 pandemic around the world. Nurses face moderate to high risks in multiple dimensions, including job stress, poor working conditions, and excessive workload [11, 12]. These risks can lead to mental health issues such as depression, burnout syndrome, and stress-related disorders [13]. The COVID-19 pandemic has exacerbated these risks, with nurses reporting high emotional work and workload [14]. However, adequate resources, measures, and information can serve as protective factors against psychosocial risks [15]. The economic impact of mental health issues among nurses in the EU is estimated at €240 billion annually [13].

In this sense, several scales and questionnaires have been developed to assess and measure psychosocial risks, such as the Job Content Questionnaire (JCQ) [16], the Effort-Reward Imbalance Questionnaire (ERI) [17], and the Copenhagen Psychosocial Questionnaire (COPSOQ) [18]. These tools are renowned for their psychometric validity and their ability to provide valuable information on psychosocial risks in different work settings [19]. Among them, the SUSESO/ISTAS21 questionnaire, based on the second version of COPSOQ, stands out as a robust and effective tool for assessing psychosocial risks at work. This questionnaire covers a wide range of psychosocial factors, including work demands, their influence, development at work, social relationships, and emotional content [18].

The adaptation and psychometric validation of the SUSESO/ISTAS21 questionnaire in nurses in Spain, known as ISTAS_ENFERMERÍA, has resulted in a reliable and specific tool to assess psychosocial risks in this population group [20]. The results of its adaptation indicate that the ISTAS_ENFERMERÍA has adequate validity and reliability indices, which allows for a more accurate identification of the psychosocial risk factors affecting nurses. This early identification is also crucial to develop preventive and supportive interventions which will contribute to improving nurses' wellbeing and the quality of care they provide [19].

In this context, the present study aims to assess psychosocial risks in a sample of nurses in Spain.

## Methods

### Design

Descriptive cross-sectional study based on questionnaires.

### Population and Sample

The study population consisted of 325,018 nurses registered in Spain (2020 data). The scope of the study was nationwide and included nurses who met the following inclusion criteria: i) nurses who provide nursing care specific to their profession; ii) nurses residing in Spain; and iii) active nurses. Those who did not meet the inclusion criteria were excluded from the study, as well as the following: i) nurses with a recognised qualification from another country who are not yet practising in Spain; ii) professionals who do not perform nursing care functions; and iii) nurses working outside Spain.

Data were collected from nurses who worked in both Primary Care and Specialised Care for the Spanish National Health Service (NHS) or in private entities (such as care homes for the aged and private clinics, among others).

The minimum required sample was calculated to be 384 nurses, using a 5% margin of error, 95% confidence, and 50% heterogeneity. However, the total sample finally obtained was 2,765 nurses, far exceeding the minimum sample size calculated, which increased the statistical power of the study.

The sample was selected by non-probabilistic snowball sampling, starting with nurses who agreed to participate and, through dissemination, achieving the inclusion of all said participants. Participation was completely voluntary, and confidentiality of the data collected was guaranteed. All participants accepted informed consent before completing the questionnaire.

### Variables

This study examines socio-demographic, occupational, and psychosocial variables. To assess the socio-demographic profile, questions on sex, age, marital status, educational level received and completed, and province of employment were included.

As for occupational variables, firstly, aspects such as sick leave (type and frequency); number of services assigned to the professional; entity and current position held; time at current unit; employment relationship; type of contract; working hours (and its specific characteristics such as rotations, their frequency, and mode of notification); level of stress and anxiety caused by such rotation; level of job competence and how such competence affects job security; whether there are changes in the working day; whether the professional also works in another place; previous work experiences (rejection or acceptance of working contracts owing to the level of expertise); effects of lack of experience on wellbeing (sleep disturbance, anxiety, or insecurity).

The level of psychosocial risk was assessed using the ISTAS_Enfermería scale. This tool measures psychosocial factors in nursing work performance, such as work stress and quality of life at work. The validation of the tool in the nursing population obtained a Cronbach’s alpha value of 0.7. The scale presents a factorial structure of 5 dimensions with 15 items, which explain 63.6% of the variance, and it showed an optimal fit in the parameters calculated by confirmatory factor analysis (values higher than 0.90 for the TLI, NFI, and CFI, lower than 0.08 for the SRMR, and lower than 0.08 for the RMSEA [20].

The scale includes 15 items distributed in five dimensions, whose scores vary depending on the dimension: in “Support at work” and “Job satisfaction,” items are rated from 0 (Always) to 4 (Never), while in “Wellbeing at work,” “Job insecurity” and “‘Double presence,” items are rated from 4 (Always) to 0 (Never). The total score is obtained by adding the values of all items, ranging from 0 (most favourable) to 60 (least favourable), where lower scores indicate better psychosocial conditions and higher scores indicate greater psychosocial risk.

### Procedure

Data collection was conducted over a 3-month period, between 1 August and 31 October 2023, based on a digital questionnaire created using Google Forms ^©^ with questions related to the study variables and distributed through social networks and specific nursing professional groups. All participants were required to provide informed consent and explicitly confirm their professional role as nurses before being able to access the questionnaire.

The dissemination of the questionnaire was carried out by the research team, ensuring its distribution in specific nursing communities. Additionally, the Spanish General Council of Nursing supported the dissemination of the questionnaire on their official social media channels and among those registered nurses in Spain who wished to be contacted by email for research purposes.

### Data Analysis

Univariate and bivariate descriptive analyses were performed using SPSS Statistics ^©^ v26 software [21]. To assess the normality of the data, the Kolmogorov-Smirnov test was applied, which yielded a value of p < 0.05, indicating non-normality. Consequently, non-parametric tests, i.e., the Mann-Whitney U test and the Kruskal-Wallis H test, were used to compare groups, and Kendall’s Tau-B coefficient was applied to analyse correlations between variables.

To explore the relationship between the level of perceived psychosocial risk and the rest of the variables, a categorical regression analysis (CATREG) adapted to the qualitative nature of the variables was used [22]. The categorical regression analysis was performed to examine the association between psychosocial risk scores (dependent variable) and independent variables such as age, gender, marital status, educational level, and workload. The regression model included adjustments for potential confounders, and results were reported as regression coefficients (β) with 95% confidence intervals (ci). Statistical significance was set at p < 0.05. This analysis included elements of classical regression, such as the coefficient of determination (R^2^), the analysis of variance in regression, and the significance of the model parameters. The variables included in the regression model were those that showed significant differences in the bivariate analysis. The optimal scaling option in SPSS^©^ [23] was used to guarantee an adequate adaptation of the variables to the model.

### Ethical Considerations

This study was approved by the Provincial Research Ethics Committee of Huelva, Spain, with code 1520-N-23. It also complies with the guidelines established in the Declaration of Helsinki [24] and complies with the provisions of Regulation (EU) 2016/679 of the European Parliament and of the Council on the protection of individuals with regard to the processing of personal data and the free movement of such data [25], as well as with Organic Law 3/2018, of 5 December, on the Protection of Personal Data and guarantee of digital rights [26].

Data collection was carried out over a period of 3 months (01 Aug - 31 Oct, 2023), using a digital questionnaire created with Google Forms ^©^ and distributed through social networks and specific professional nursing groups, with the support of the General Council of Nursing of Spain for dissemination through its official channels and by email to registered nurses interested in participating in research. This questionnaire included questions related to the study variables, and all participants were required to provide informed consent, explicitly confirm their professional role as nurses, and tick the appropriate box after reading the information provided about the study and the informed consent document. Rigorous measures were implemented to ensure the privacy and confidentiality of the participants, in accordance with the provisions of the Organic Law on Data Protection and Guarantee of Digital Rights [26], with data being stored in a protected database accessible only to the research team.

## Results

### Descriptive Analysis

The sample consisted of 2,765 nurses from all over Spain, with a mean age of 40.87 years. Of the total sample, 87.3% were women, while only 12.3% were men and a small percentage identified themselves as non-binary or preferred not to state their sex. The mean ISTAS21 score was 1.78 (SD = 0.503), with no significant differences between sexes (p = 0.503). Tables 1, 2 describe the main results of the descriptive bivariate analysis for socio-demographic and occupational variables.

**TABLE 1:** Socio-demographic variables (Data from the ISTAS_Enfermería Study, Spain, 2023).

Variables	Total sample (n = 2,765)	ISTAS21 mean score (δ/SD)	Contrast of hypotheses
Sex			
Male	341 (12.3%)	1.792 (SD = 0.562)	P = 0.503^a^
Female	2,414 (87.3%)	1.779 (SD = 0.494)	
Non-binary	1 (0.0%)	1.200 (SD = 0)	
Rather not say	9 (0.3%)	1.874 (SD = 0.590)	
Age			
Mean	40.87 years	1.78 (SD = 0.5)	**Tau b** ^b^ **= -0.12 (p 0.001)**
Marital status			
Married	1,186 (42.9%)	1.722 (SD = 0.494)	**0.001** ^a^
Divorced	117 (4.2%)	1.751 (SD = 0.500)	
With a partner (cohabiting)	748 (27.1%)	1.840 (SD = 0.517)	
With a partner (non-cohabiting)	242 (8.8%)	1.828 (SD = 0.484)	
Separated	41 (1.5%)	1.824 (SD = 0.561)	
Single	422 (15.3%)	1.819 (SD = 0.492)	
Widowed	9 (0.3%)	1.814 (SD = 0.546)	
Educational level			
Degree	588 (21.3%)	1.809 (SD = 0.480)	**0.007** ^a^
Former Higher degree	42 (1.5%)	1.820 (SD = 0.550)	
Former Lower degree	704 (25.5%)	1.722 (SD = 0.464)	
Doctorate	59 (2.1%)	1.714 (SD = 0.641)	
Specialty	397 (14.4%)	1.751 (SD = 0.532)	
Master’s degree	664 (24%)	1.831 (SD = 0.522)	
Expert	298 (10.8%)	1.803 (SD = 0.504)	
Vocational training	13 (0.5%)	1.764 (SD = 0.493)	

δ Mean score.

SD, standard deviation.

^a^ Kruskal Wallis H test.

^b^ Kendall’s Tau-B.

**TABLE 2:** Occupational variables (Data from the ISTAS_Enfermería Study, Spain, 2023).

Variables	Total sample (n = 2,765)	ISTAS21 mean score (δ/SD)	Contrast of hypotheses
Employment relationship			
I am a civil servant, statutory employee	911 (32.9%)	1.640 (SD = 0.466)	**0.001** ^a^
I am an interim	713 (25.8%)	1.859 (SD = 0.496)	
I am a permanent employee (I have a permanent contract, …)	466 (16.9%)	1.740 (SD = 0.486)	
I have a permanent discontinuous contract	43 (1.6%)	1.965 (SD = 0.605)	
I am a temporary employee with a training contract (temporary contract under training)	41 (1.5%)	1.660 (SD = 0.576)	
I am temporary employee (contract for limited work and services, circumstances of production, …)	561 (20.3%)	1.920 (SD = 0.499)	
Without a contract	30 (1.1%)	2.164 (SD = 0.512)	
Type of contract			
Full-time	2,403 (86.9%)	1.777 (SD = 0.508)	0.614^a^
Full-time with reduced working hours (maternity/paternity, studies, disability)	193 (7.0%)	1.806 (SD = 0.454)	
Part-time	113 (4.1%)	1.788 (SD = 0.500)	
Part-time with reduced working hours (maternity/paternity, studies, disability)	56 (2.0%)	1.839 (SD = 0.429)	
Number of services			
Less than 5 different services	564 (23.7%)	1.672 (SD = 0.489)	**0.001** ^a^
Between 5 and 10 different services	1,025 (37.1%)	1.750 (SD = 0.495)	
More than 10 different services	1,084 (39.2%)	1.876 (SD = 0.502)	
Work area			
Urgent and emergency care	21 (0.8%)	1.920 (SD = 0.437)	**0.001** ^a^
Management	3 (0.1%)	1.155 (SD = 0.443)	
General management, governing, and administration	7 (0.3%)	1.714 (SD = 0.707)	
Educational field	18 (0.7%)	1.844 (SD = 0.698)	
Primary Care	570 (20.8%)	1.729 (SD = 0.534)	
Teaching and research	12 (0.4%)	1.522 (SD = 0.620)	
Public hospital	1710 (62.3%)	1.785 (SD = 0.481)	
Private hospital	199 (7.3%)	1.764 (SD = 0.497)	
Various entities	135 (4.9%)	1.923 (SD = 0.530)	
Subsidised hospital	20 (0.7%)	1.730 (SD = 0.397)	
Other entities not related to nursing	5 (0.2%)	2.053 (SD = 0.536)	
Public bodies	8 (0.3%)	1.675 (SD = 0.560)	
Not currently working	36 (1.3%)	2.085 (SD = 0.557)	
Current position			
Assistant Nurse	2,287 (82.7%)	1.804 (SD = 0.493)	**0.001** ^a^
Resident Nurse	37 (1.3%)	1.690 (SD = 0.551)	
Nurse in charge of unit or service without recognised management position	139 (5%)	1.711 (SD = 0.477)	
Unit or area supervisor with management functions only	93 (3.4%)	1.465 (SD = 0.455)	
Unit supervisor with direct patient care as well as management functions	84 (3%)	1.590 (SD = 0.517)	
Specialist nurse	29 (1%)	1.671 (SD = 0.602)	
School nurse	11 (0.4%)	1.636 (SD = 0.598)	
Teaching and research	9 (0.3%)	1.837 (SD = 0.501)	
Time at current unit			
Unemployed	32 (1.2%)	2.143 (SD = 0.498)	**0.001** ^a^
Less than 30 days	193 (7.0%)	1.969 (SD = 0.501)	
1–6 months inclusive	446 (16.1%)	1.848 (SD = 0.528)	
6 months to 2 years inclusive	570 (20.6%)	1.803 (SD = 0.505)	
Between 2 and 5 years inclusive	615 (22.2%)	1.765 (SD = 0.496)	
Between 5 and up to 10 years inclusive	337 (12.2%)	1.744 (SD = 0.486)	
More than 10 years	572 (20.7%)	1.662 (SD = 0.461)	
Sick leave			
I have not had any sick leave in the last year	1,444 (52.3%)	1.689 (SD = 0.494)	**0.001** ^a^
I have had ONE sick leave in the last year	1,028 (37.2%)	1.842 (SD = 0.484)	
I have had TWO OR MORE sick leaves in the last year	280 (10.1%)	2.027 (SD = 0.507)	
Prolonged/long-term sick leave (more than 1 year)	9 (0.3%)	1.837 (SD = 0.433)	
Leave of absence	2 (0.1%)	1.766 (SD = 0.141)	
Type of sick leave			
Sick leave due to an accident at work	46 (3.9%)	1.891 (SD = 0.551)	0.991^a^
Sick leave due to occupational disease	102 (8.6%)	1.883 (SD = 0.481)	
Paternity/maternity leave	97 (8.2%)	1.854 (SD = 0.483)	
Sick leave due to common illness	940 (79.3%)	1.895 (SD = 0.496)	

δ Mean score.

SD, standard deviation.

^a^ Kruskal Wallis H test.

The results show significant differences for the marital status, educational level, and province of work variables concerning the ISTAS_Enfermería scores and the different categories of the variable. Married people and those with a doctorate or degree had lower scores (indicating better psychosocial conditions) than those with a master’s degree.

Age reveals a negative and significant relationship with the scores (Tau b = −0.12, P < 0.001), suggesting a lower perception of risk with increasing age. On the other hand, no significant differences were found based on sex (P = 0.503), although there was a greater proportion of women in the sample (87.3%).

The mean ISTAS_Enfermería score showed significant differences according to employment relationship, number of services, work area, current position, time at current unit, and sick leave (p = 0.001). Higher values were found in respondents who were working without a contract (mean = 2.164), were unemployed (mean = 2.143), or had had two or more sick leaves in the previous year (mean = 2.027), while the lowest scores were associated with civil servants/statutory employees (mean = 1.640), managers (mean = 1.155), and those who had been in the same unit for more than 10 years (mean = 1.662). There were no significant differences according to the type of contract or the reason for sick leave.

### Multivariate Regression Analysis

A categorical regression analysis was carried out using the ISTAS_Enfermería scale total score as the dependent variable and the following variables as predictors: age; marital status; educational level; employment relationship; number of services; current position; time at current unit; and sick leave. The model showed a coefficient of determination of R^2^ = 0.095 (adjusted R^2^ = 0.089) and an overall significance of p < 0.001, indicating that the model was of low but significant explanatory power.

The variables that contributed significantly to the model were: age; education completed; employment relationship; number of services; time at current unit; and sick leave (Table 3). In addition, the results indicate that for each additional year, professionals perceive 0.12 times lower levels of psychosocial risk (β = −0.124), suggesting that younger nurses are more susceptible to stress. Meanwhile, professionals without a contract experience 0.063 times higher levels of psychosocial risk than civil servants (β = 0.063), while those who are assigned to more than ten rotating services perceive 0.095 times higher levels of psychosocial risk than those with less rotation (β = −0.095). In the same way, nurses engaged in nursing care reported risk levels 0.065 times higher than supervisors and managers (β = −0.065), who face less direct exposure to stressors. Finally, professionals with two or more sick leaves in the previous year reported 0.16 times higher levels of psychosocial risk than those with no sick leaves (β = −0.162).

**TABLE 3:** Model fit and significance of regression analysis (Data from the ISTAS_Enfermería Study, Spain, 2023).

R^2^ = 0.095^a^	Fisher’s F = 32.13		p < 0.001	
Variable	Coefficient (β)	Degrees of freedom	F-statistic	p value
Age	−0.124	2	28.915	<0.001
Educational level	0.064	2	7.448	<0.001
Employment relationship	0.063	1	4.179	0.041
Number of services	−0.095	1	9.185	0.002
Current position	−0.065	2	10.857	<0.001
Time at current unit	0.053	3	3.656	0.012
Sick leave	−0.162	2	72.631	<0.001

^a^ Regression coefficient.

Despite the low explanatory power of the model, the results reveal significant relationships between several independent variables and the ISTAS_Enfermería scale score. This highlights the relevance of socio-demographic and occupational factors in the assessment.

## Discussion

Psychosocial risks in the work environment represent a major challenge for the health and wellbeing of workers. Particularly in highly demanding professions such as nursing, these risks become more prominent due to factors such as emotional burden, continuous stress, and interaction with patients under high-pressure conditions. A study on the mental health of healthcare workers during the COVID-19 pandemic found high levels of emotional exhaustion, stress, and anxiety among nurses, highlighting the prevalence of psychosocial risks linked to their work environment. The nursing environment is characterised by high emotional demands and constant pressure to respond effectively in difficult situations [27]. In the same line, another article had noted that the characteristics of the nursing job, such as prolonged exposure to human suffering, intense emotional burden, and high work demands, significantly increase psychosocial risks [15]. This context underlines the need to implement effective interventions to effectively address these factors and protect the mental and physical health of health professionals.

The present analysis addresses the results obtained in the assessment of psychosocial risks using the ISTAS_Enfermería scale in this population group. The main findings revealed a mean score of 1.78 on the scale, reflecting a low to moderate level of risk. However, this score overlooks significant differences linked to factors such as employment relationship, marital status, educational level, number of services assigned to the professional, time in the unit, and sick leave. All this indicates that specific job characteristics, such as the diversity of assigned areas and job stability, are determinants for understanding the perception of the working environment beyond socio-demographic variables.

Although the analysis identified significant relationships between certain socio-demographic variables and psychosocial risks, the explanatory model showed a low predictive capacity (R^2^ = 0.095). This suggests that, while factors such as age, marital status, and educational level seem to influence the scores obtained, there are other elements that have not been considered and that could be playing a significant role in the variations observed. According to the literature, despite socio-demographic variables being significant, other structural and contextual factors such as workload, organisational culture, and perceived social support also have a critical impact on the experience of psychosocial risks [28]. For example, variables such as leadership quality, perceived fairness at work, and team cohesion have proven particularly influential, suggesting that explanatory models need to incorporate these dimensions to fully understand how psychosocial risks are shaped [29].

Among the most notable findings is the negative relationship between age and the perception of psychosocial risks. This result, consistent with previous research, suggests that younger workers face greater difficulties in adapting to work demands, possibly owing to a lack of professional experience or stability, in contrast to their more experienced counterparts. According to Hsu [30], young workers report higher levels of work-related stress and emotional exhaustion than their more experienced peers, who tend to benefit from greater resilience and resources accumulated throughout their careers. Interventions to improve coping skills and resilience in younger workers, such as mentoring and skill development programmes, can be instrumental in reducing this vulnerability.

Regarding the sex of the participants, no significant differences were found in relation to the perception of psychosocial risks. This finding is consistent with research such as the one by Méndez-Rivero et al. [31], who found that, in homogeneous work environments in terms of working conditions, differences between men and women in the perception of psychosocial risks were found to be minimal, suggesting that risks can equally affect both sexes when working conditions are similar. However, this study also suggests that occupational segregation and specific work dynamics may influence how these risks are experienced, especially in contexts of job insecurity, underlining the need to consider broader organisational and contextual factors.

In addition, Cattani and Rizza [32] explored how occupational roles influenced by sex may modify exposure to psychosocial risks, highlighting that the representation of women is often greater in sectors with high emotional demands, such as education and health. While not always translating into significant differences in terms of perception, this factor could add relevant nuances in certain work settings [32]. Therefore, although the data from this analysis did not reveal significant differences by sex, previous research indicates that there are contexts where such differences may be apparent depending on the occupational structure and the responsibilities assigned.

Marital status also emerged as a significant factor in this analysis, with married people scoring lower on psychosocial risk. This pattern is consistent with previous research indicating that personal stability and social support at home both have a protective effect in the face of work demands. Another study has already highlighted that the emotional and practical support offered by a partner or support network could reduce stress levels and lead to more effective coping strategies, especially in highly demanding professions such as nursing [33].

Conversely, educational level exhibited an inverse trend, as a higher number of nurses with advanced degrees, such as master’s degrees, reported increased levels of psychosocial risk, as reflected in their higher scores. This suggests that despite their educational attainment, these individuals may experience greater work-related psychosocial demands or stressors. These findings align with studies indicating that the greater responsibilities and expectations associated with highly qualified profiles heighten the perception of work pressure and workload [34].

Overall, the results show that socio-demographic variables provide relevant information on psychosocial risks in the nursing profession, but are insufficient to fully explain variances. In this regard, it is crucial to address work, organisational, and group factors, given that individual characteristics must be analysed in conjunction with the work context.

Job stability plays a key role in the perception of psychosocial risks. Workers with temporary contracts or no contract reported higher levels of stress and psychosocial risks compared to those with stable or permanent jobs, such as civil servants [35]. This is in line with studies indicating that job insecurity increases anxiety, negatively affecting employees’ mental and emotional health. Besides, the perception of job stability improves economic security and contributes to greater organisational identification, which reinforces resilience in the face of work demands [36]. These results underscore that job insecurity exacerbates psychosocial risks by reducing the sense of control and predictability over one’s future at work, which constitute fundamental aspects of good occupational health. The number of services professionals were assigned to showed a significant relationship with risk scores, being higher among those assigned to multiple services. Several studies have explored the relationship between workload and stress in nursing professionals, highlighting how multi-service responsibilities can intensify these risks. It has been observed that a significant percentage of nurses experience high levels of workload and stress associated with the diversity of work areas and the lack of continuity in duties [37]. Recent research has also shown that increased workload, especially in terms of mental demands, generates difficulties in establishing stable routines and increases stress levels among care and administrative staff [38]. These results underline the importance of considering the impact of multi-service assignment on the occurrence of psychosocial risks and on the mental health of professionals.

High-pressure work areas, such as emergency departments, are associated with high levels of psychosocial risk due to the intensity of demands and constant exposure to critical situations. These environments generate high levels of stress due to factors such as emotional overload, the need to make quick and accurate decisions, and the pressure to meet strict deadlines [5, 39]. Such a combination of cognitive and emotional demands in a high-demand context can lead to significant psychosocial vulnerability, affecting workers' mental health. Also, unavailability of adequate resources and limited support in these environments increase stress, which may in turn result in work-related health problems such as burnout and anxiety [5, 40].

Sick leave is significantly associated with high levels of psychosocial risk, which can be interpreted in two ways: as a direct consequence of exposure to adverse work environments or as an early indicator of pre-leave stress and burnout. Research suggests that chronic stress, especially in high-demand settings such as healthcare, can lead to physical and emotional overload, resulting in absences from work [41]. Moreover, the effort-reward model reinforces the idea that lack of resources in the face of high work demands contributes to workers' burnout, which can then translate into sick leave [42]. This underlines the need to address psychosocial factors at an early stage to prevent more serious negative effects.

Overall, the assessment of these work-related variables reflects how specific work conditions can exacerbate or mitigate psychosocial risks. This finding underscores the importance of organisational interventions that promote more stable and sustainable environments for nursing professionals.

This study has several strengths that highlight its contribution to the field of psychosocial risk research in nursing. These include the use of a significantly large sample (n = 2,765), which allows for a robust analysis and improves the generalisability of the results. Furthermore, the adaptation and psychometric validation of the ISTAS_Enfermería questionnaire specifically for nurses in Spain is a key methodological strength, as it provides a reliable tool for measuring psychosocial risks in this context. Another strength is that the sample comprises 2,765 nurses, of whom 85.5% identify as female and 14.5% as male. This distribution mirrors national statistics, which indicate that 85.5% of nurses in Spain are female and 14.5% are male [43]. Furthermore, available data show that 53.3% of nurses in Spain are under 45 years old [43]. Although the study did not collect specific age data, the reported levels of professional experience among our participants suggest a similar age distribution. These similarities in gender and inferred age profile indicate that the sample provides a representative cross-section of the Spanish nursing workforce. Consequently, the results of our study can be reasonably extrapolated to the broader nursing population in Spain.

However, this study has certain limitations that should be considered. Firstly, the cross-sectional design limits the ability to establish causal relationships between the examined variables, restricting interpretation to the observed associations. Secondly, although the total sample greatly exceeded the minimum sample size calculated, the non-probabilistic snowball sampling may have introduced selection biases, which could affect the representativeness of the results and limit their generalisability at the national level. Also, although the applied questionnaire had been previously validated, the use of self-report instruments may involve subjectivity and possible social desirability bias in the participants’ responses. It should also be noted that the predominance of women in the sample (87.3%) could lead to a bias in the interpretation of possible sex differences, although the size of the sub-samples was sufficient for statistically robust analyses. Finally, it would be important to complement these findings with further qualitative studies that explore participants' experiences and perspectives in greater depth, as well as longitudinal research that allows for analysis of changes over time.

To further elaborate on the results, the findings of this study may have implications for the management of psychosocial risks in the field of nursing. The identification of occupational and socio-demographic factors associated with higher levels of psychosocial risks provides a solid basis for designing specific and tailored interventions aimed at improving working conditions. For example, the results highlight the need to address job insecurity and sick leave as priorities in the management of nurses' wellbeing. Additionally, the data obtained can serve to inform institutional policies that promote job stability, professional development, and strategies to mitigate stress and anxiety in this population group. On a practical level, the results stress the importance of implementing preventive and supportive programmes that not only benefit the health of nurses, but also improve the quality of care they provide to patients, thus contributing to the strengthening of the healthcare system in general.

### Conclusion

The findings indicate that higher educational levels and greater workload are linked to higher psychosocial risk, while marriage and older age seem to have a protective effect. These results highlight the need for targeted interventions to mitigate work-related psychosocial stressors. Organisational strategies such as reducing workload, improving work-life balance, and providing adequate support for employees with higher education levels could help alleviate these risks. Future studies should focus on identifying specific mechanisms through which educational level influences psychosocial risk and developing evidence-based interventions tailored to different professional groups.
